# Supplementary material for: All-Trans Retinoic Acid Fosters the Multifarious U87MG Cell Line as a Model of Glioblastoma
Source: Brain Sci. 2021 Jun 18;11(6):812. doi: 10.3390/brainsci11060812 (PMC8234004; doi:10.3390/brainsci11060812)
Supplement: Supplementary file 1 [file brainsci-11-00812-s001.zip › brainsci-1256632-supplementary.pdf]

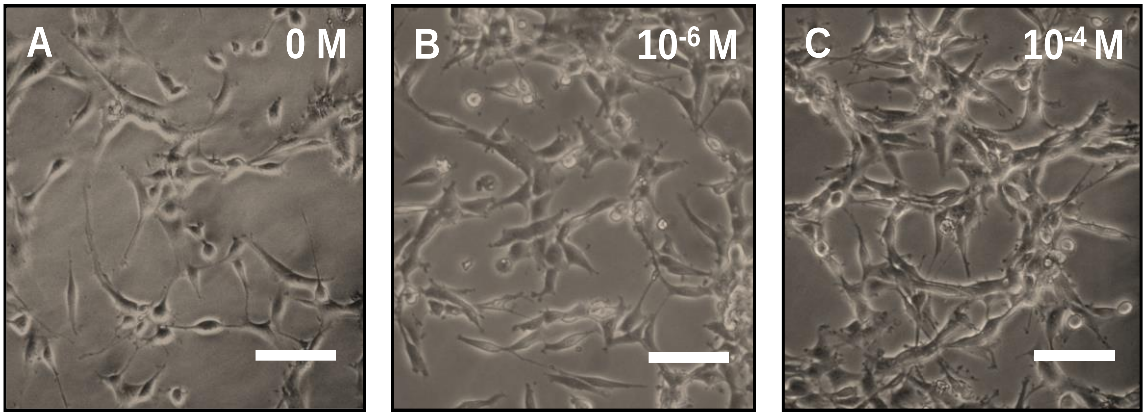

Supplementary Figure 1 (supporting Figure 1): Representative Bright field images of the glioblastoma cell line U87MG (ECACC 89081402) treated for 7 days with all-trans retinoic acid (ATRA). The ATRA concentration is indicated in each panel (upper right). Scale bar 100 μm.

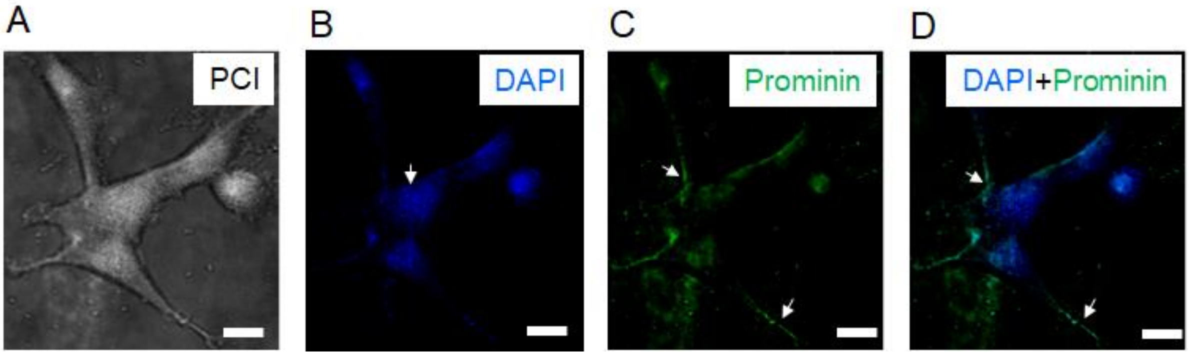

Supplementary Figure 2 (supporting Figure 1): The stem cell marker Prominin-1 shows low levels of expression in U87MG (ECACC 89081402) after 7 days treatment with ATRA (10^{-4} M). (A) Phase contrast image (PCI); (B) DAPI stained nuclei (blue); (C) Prominin-1 stained cells (green); (D) Merged image. Arrows indicate presence of Prominin-1. Scale bar 20 μm.

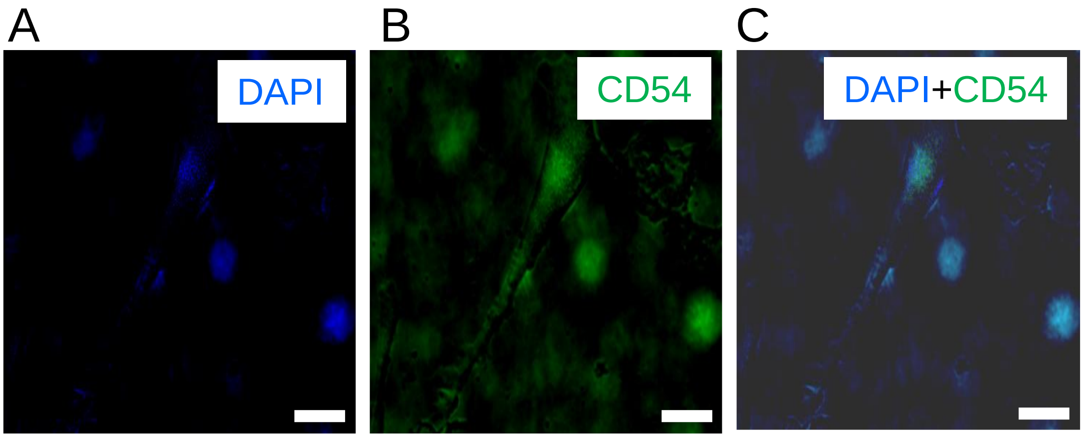

Supplementary Figure 3 (supporting Figure 2): Intercellular adhesion molecule 1 (ICAM-1/CD54) shows low levels of expression in control U87MG (ECACC 89081402) not treated with ATRA. (A) DAPI stained nuclei (blue); (B) CD54 stained cells (green); (C) Merged image. Scale bar 40 μm.
